# Dispersing away from bad genotypes: the evolution of Fitness-Associated Dispersal (FAD) in homogeneous environments

**DOI:** 10.1186/1471-2148-13-125

**Published:** 2013-06-19

**Authors:** Ariel Gueijman, Amir Ayali, Yoav Ram, Lilach Hadany

**Affiliations:** 1 Department of Molecular Biology and Ecology of Plants, Tel Aviv University, Tel-Aviv 69978, Israel; 2 Department of Zoology, Tel Aviv University, Tel-Aviv 69978, Israel

**Keywords:** Phenotypic plasticity, Genetic mixing, Outcrossing, Stress-induced variation, Fitness-dependent dispersal, Condition-dependent dispersal, Partial migration, Stochastic simulations

## Abstract

**Background:**

Dispersal is a major factor in ecological and evolutionary dynamics. Although empirical evidence shows that the tendency to disperse varies among individuals in many organisms, the evolution of dispersal patterns is not fully understood. Previous theoretical studies have shown that condition-dependent dispersal may evolve as a means to move to a different environment when environments are heterogeneous in space or in time. However, dispersal is also a means to genetically diversify offspring, a genetic advantage that might be particularly important when the individual fitness is low. We suggest that plasticity in dispersal, in which fit individuals are less likely to disperse (Fitness-Associated Dispersal, or FAD), can evolve due to its evolutionary advantages even when the environment is homogeneous and stable, kin competition is weak, and the cost of dispersal is high.

**Results:**

Using stochastic simulations we show that throughout the parameter range, selection favors FAD over uniform dispersal (in which all individuals disperse with equal probability). FAD also has significant long-term effects on the mean fitness and genotypic variance of the population.

**Conclusions:**

We show that FAD evolves under a very wide parameter range, regardless of its effects on the population mean fitness. We predict that individuals of low quality will have an increased tendency for dispersal, even when the environment is homogeneous, there is no direct competition with neighbors, and dispersal carries significant costs.

## Background

Dispersal affects fundamental processes in ecology and evolution such as gene flow, genetic drift, and inbreeding. However, the evolution and regulation of dispersal are not yet fully understood. Dispersing individuals spend time and energy and, among other costs, may be exposed to uninhabitable environments, predation, and the risk of failing to find a new site in time for reproduction [1]. Classical theory suggests that individuals may disperse in order to change their environment [2-4], to avoid competition with kin [5-8], to avoid inbreeding [9-11], or a combination of the above [12-16]. These models, however, do not account for individual plasticity in dispersal behavior, in particular the ability of an individual to adjust its dispersal behavior according to circumstances (biotic, abiotic, internal, external, or a combination of them). Such plastic dispersal has been observed in many species (reviewed in [17-20]; Table 1).

**Table 1:** Empirical evidence of plastic dispersal

**Citation**	**Species**	**Data**	**Dispersal driver supported**
Baguette et al., 2011 [21]	Bog fritillary butterfly (*Boloria eunomia*)	Lower habitat quality raises the emigration rates and higher habitat quality raises the residence probability	Negative density-dependence as a cue; lower habitat quality (limited resources)
Vercken et al., 2012 [22]	Juvenile common lizard (*Lacerta vivipara)*	Frequencies of female classes affect dispersal decisions differentially among classes	Competition with superior conspecifics; conspecifics as environmental condition cue
Donohue, 2003[23], Imbert & Ronce, 2001 [24]	Holy’s Hawk’s-beard (*Crepis sancta*)	Environmental stress results in a higher proportion of wind-dispersal structures	Lower habitat quality (limited resources)
Wender et al., 2005 [25]	*Arabidopsis thaliana*	Density effects on maternal traits, such as plant height and fruits, have diverse effects on seed dispersal patterns	Density-dependence (with various effects)
Hanski et al., 1991 [26]	Siberian flying squirrels (*Sorex araneus*)	Juvenile dispersal strategy changes with density from conditional to effectively non-conditional	Competition with superior individuals; density-dependence
Chaput-Bardy et al., 2010 [27]	Damselfly (*Calopteryx splendens*)	Females tend to disperse more often than males; emigration probability decreases with density; probability to move decreases when sex-ratio is male biased	Conspecific negative density-dependence (sex-ratio dependence); sex-dependence;
Clarke et al., 2008 [28]	Chacma baboons (*Papio hamadryas ursinus*)	Males disperse; individual well-being combined with numbers of males and females is associated with differential and plastic dispersal strategies	Competition with conspecifics
Solmsen et al., 2011 [29]	African striped mice (*Rhabdomys pumilio*)	Locally inferior males disperse with a higher tendency	Competition with conspecifics; sex-dependence(?)
Shafer et al., 2011 [30]	The mountain goat (*Oreamnos americanus*)	Dispersers have lower observed heterozygosity compared to their population of origin	Inbreeding avoidance; heterosis; competition with conspecifics

Recently, various ecological drivers of plastic dispersal strategies have been suggested [17,18,31,32]. Plastic migration can be induced, among other causes, by density effects [33-36], by competition with superior individuals [37,38], by limited resources, e.g. nutrients [24], or by predation risk [39,40]. In all these models, the dispersing individual is assumed to benefit directly from dispersal by moving to a different environment: one where there is a chance to find fewer or less intense competitors, more tolerable conditions, more food, or fewer predators [20]. For such a mode of dispersal to be advantageous, the expected benefits resulting from moving to the new location should be greater than the expected cost of dispersal for the individual [17,41]. Consequently, one would not expect the evolution of plastic migration in a uniform and stable environment where the cost of dispersal is high, and sib competition is weak.

Here we concentrate on the effect of dispersal on the rate of outcrossing. Outcrossing has been suggested in the past as a possible advantage of dispersal in general (heterosis, e.g. [42,43]), and we suggest that it favors the evolution of plastic dispersal in particular. From the point of view of a gene regulating dispersal tendency, dispersal can be viewed as a move in both the physical and the genotypic space, because a dispersing individual has a higher probability of mixing its genome with that of an unrelated individual. Both types of movement are more beneficial for a gene linked to a maladapted genetic background than to one linked to a well-adapted one. In particular, even in the absence of environmental heterogeneity, and even if deleterious mutations are not recessive, selection can favor a gene inducing increased dispersal tendency among the less fit individuals. Thus dispersal may be regulated according to the well-being of the individual, so that fit individuals are less likely to disperse, i.e. Fitness-Associated Dispersal, or FAD [44]. Indeed, such association between poor condition and dispersal has been observed in many organisms [24,30,45-47] (but not always, e.g. [48]). Different terms, including condition-dependent dispersal and matching habitat [17,49], are used in the literature to describe such associations. Since we concentrate on the genetic (internal) aspects of individual condition rather than the environmental (external) ones, we use FAD, which has been used in the past in the exact same sense [44].

In this work, we used stochastic simulations to study the evolutionary dynamics of a modifier gene that determines the individual dispersal strategy in an homogeneous environment. We show that FAD can evolve even when the environment is homogeneous and the cost of dispersal is high. We predict that stress will result in an increased tendency to disperse even when the cost of dispersal is very high, as long as there is potential for regulated plasticity in dispersal rates.

## Results

**Figure 1:**
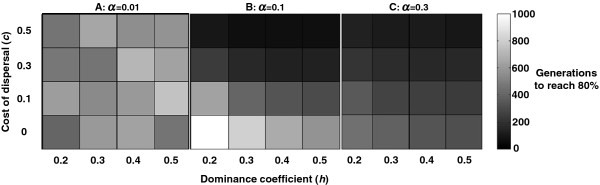
**Invasion of Fitness-Associated Dispersal.** FAD invaded a population with a uniform dispersal rate throughout the parameter range tested (p ≤ 10^-10^ for each parameter set, exact binomial test), even though the average dispersal rate of the FAD and uniform subpopulations was held equal in each simulation (*α*_*F*_ = *α*_*U*_). The figure shows the mean number of generations required for a FAD allele to increase in frequency from 0.01 to 0.8 in the cases where FAD took over the population (cells’ clarity increases with number of generations), for different values of the mean dispersal rate *α* (**A**: *α* = 0.01, **B**: *α* = 0.1, **C**: *α* = 0.3). As the cost of dispersal increases the relative advantage of fit FAD individuals that rarely disperse increases and it takes FAD less time to spread. As the average rate of dispersal increases (from panel **A** to **C**), FAD takeover occurs more rapidly when the dispersal cost is high, and more slowly when it is low. The effect of the dominance coefficient *h* was notable mostly at high dispersal rates together with low dispersal cost, where the number of heterozygotes was highest. Parameters: N ≥ 900 for *α* = 0.01; N ≥ 100 for all other values of *α*.

**Figure 2:**
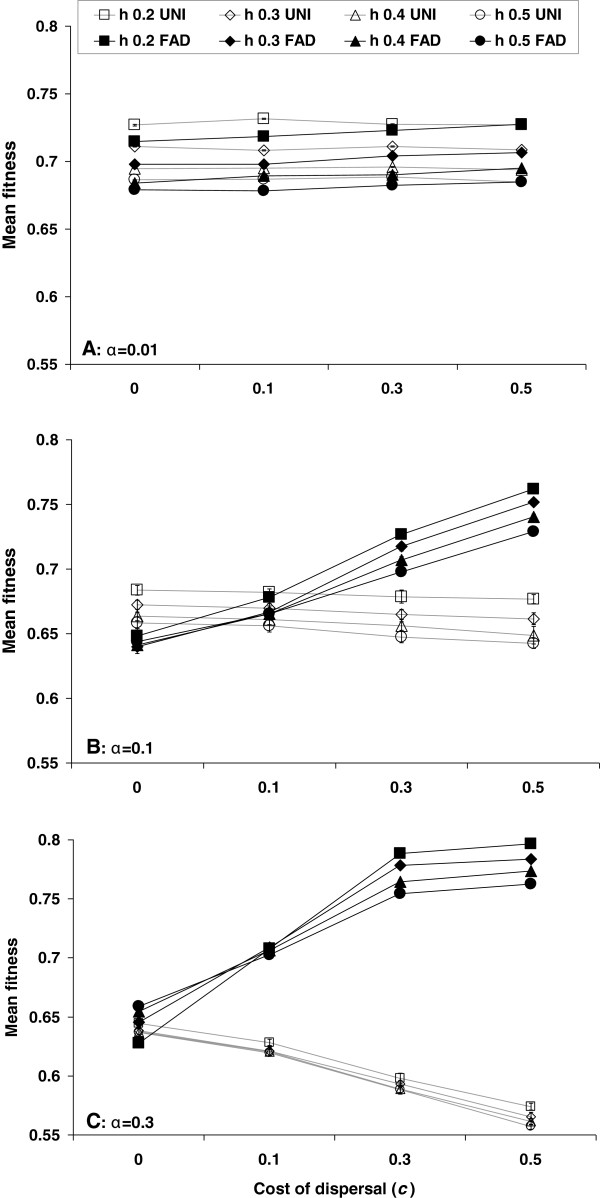
**Mean fitness under different dispersal rules.** Mean fitness (±SE) at the steady state in populations homogeneous at the modifier locus as a function of the cost of dispersal *c*, the dominance coefficient *h*, and the mean dispersal rate *α* (**A**: *α* = 0.01, **B**: *α* = 0.1, **C**: *α* = 0.3). The mean fitness of UNI (open markers) decreases with the cost, because the cost is a component of the fitness. This effect becomes stronger as the dispersal rate *α* increases (from **A** to **C**) and the cost is paid more often. In contrast, the mean fitness of FAD (filled markers) increases with the cost of dispersal, because less fit individuals pay the cost more often and deleterious alleles are purged from the population. As the dominance coefficient *h* increases, the masking of deleterious mutations in a heterozygous state weakens and fitness is slightly reduced in most cases. Note that in most cases the error bars, showing the standard error of the mean, are smaller than the markers.

We found that Fitness-Associated Dispersal (FAD) is favored by selection over uniform dispersal throughout the examined parameter range. Specifically, when alleles for FAD invaded UNI populations with the same average dispersal rate, invasion was successful throughout the parameter range (Figure 1, p < 10^-10^, exact binomial test). Takeover time generally decreased with the cost of dispersal, since the relative advantage of the high-quality FAD genomes (which were dispersing less often) was greater than that of the high quality UNI genomes (Figure 1). This trend was mainly noticeable for high values of the dispersal rate, since in this case the cost of dispersal is taken into consideration more often. However, for a low cost of dispersal the takeover time actually increased with dispersal rate, since in this case FAD leads to slightly decreased mean fitness (Figure 2).

**Figure 3:**
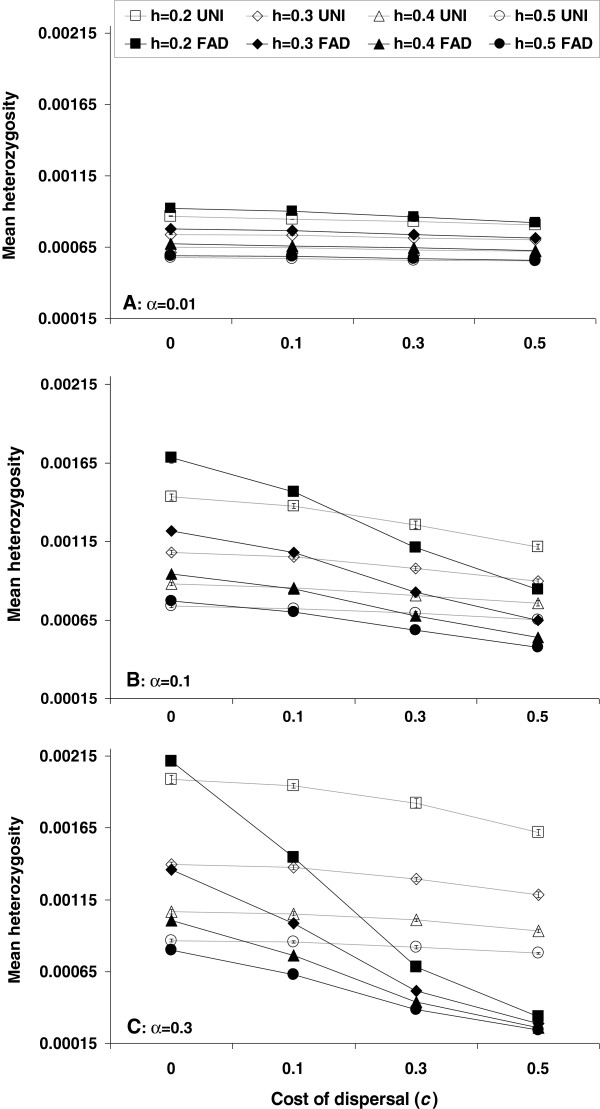
**Mean heterozygosity under different dispersal rules.** Mean frequency (±SE) of deleterious heterozygous alleles at the steady state in populations homogeneous at the modifier locus - either FAD allele (filled markers) or UNI allele (open markers). Heterozygosity is plotted as a function of the cost of dispersal *c*, of the dominance coefficient *h*, and of the mean dispersal rate (**A**: *α* = 0.01, **B**: *α* = 0.1, **C**: *α* = 0.3). Heterozygosity decreases as *h* increases in all cases, because the masking of deleterious mutations in a heterozygous state is less efficient. Under both dispersal rules heterozygosity decreases with *c,* because fewer dispersers - which are likely to be outcrossers - survive; but the effect is stronger under FAD, because under FAD individuals carrying many deleterious mutations are more likely to disperse and pay the cost of dispersal. Comparing Figures 2 and 3, heterozygosity with FAD tends to be higher when mean fitness is lower, but not always to the same extent (see, for example, the effect of *c* in Figure 2C compared with Figure 3C). Note that in most cases the error bars, showing the standard error of the mean, are smaller than the markers.

In our model, fitness and heterozygosity are two distinct concepts – heterozygosity is defined by the number of loci in heterozygote state and fitness is defined by the number of loci both in a heterozygote state and in a homozygote state, according to the dominance coefficient *h* and the selection coefficient *s*. The interaction of these two key concepts with FAD is complex and depends on the cost of dispersal. Under FAD, the cost is paid mainly by individuals with a higher than average number of deleterious mutations, reducing their ability to reproduce. Therefore, when the cost is high, deleterious mutations are purged from the population more effectively with FAD than with UNI. This purging effect drives an increase in the population mean fitness (Figure 2) and a reduction in the mean heterozygosity of deleterious mutations (Figure 3). When the dispersal rate increases, more individuals pay the cost of dispersal and the purging effect becomes stronger (compare panels A and C in Figure 2).

Under a low cost of dispersal with FAD, unfit individuals disperse and outcross more often than their fit counterparts, leading to a more even distribution of deleterious mutations in the population, and less effective natural selection. As a result, population mean fitness decreases (Figure 2), while mean heterozygosity of deleterious mutations increases (Figure 3). Mean heterozygosity of neutral mutations might also increase [50-52], and heterozygosity could be advantageous in the very long term if the environmental conditions change [53,54]. The effect of dispersal on the distribution of deleterious alleles is stronger than that of random genetic drift – when the cost of dispersal was removed entirely (*c* = 0), a higher dispersal rate, which leads to a larger effective population size, was correlated with a reduced population mean fitness (Figure 2).

**Figure 4:**
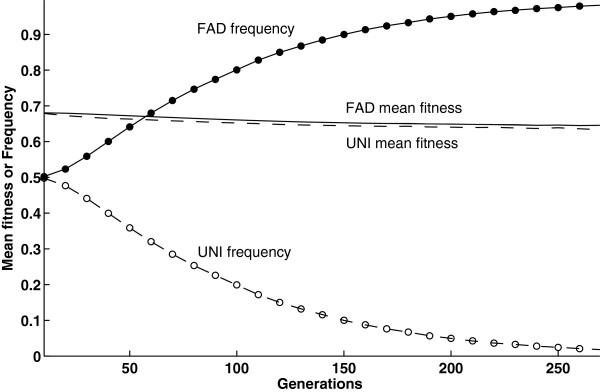
**FAD can invade despite a long-term disadvantage.** The figure shows the frequency and mean fitness of FAD (filled markers solid line and solid line, respectively) and UNI (open markers dashed line and dashed line, respectively) modifier alleles in an average of 100 runs as a function of time. For this parameter set, FAD succeeds in invading a population with a uniform dispersal rate (with equal average dispersal rates, *α*_*F*_ = *α*_*U*_), but the invasion is accompanied by a substantial decrease in mean fitness for both the FAD and the UNI sub-populations. FAD invasion successfully invades because of the *abandon-ship* advantage: FAD modifier alleles tend to break away more effectively from deleterious genetic backgrounds through dispersal and outcrossing. They become associated with relatively good genetic backgrounds, consistently leading to higher mean fitness than UNI throughout the period of invasion. Parameters: cost of dispersal *c* = 0, dominance coefficient *h* = 0.2, average dispersal rate *α*_*F*_ = *α*_*U*_ =0.1. Qualitatively similar results were obtained for the introduction of FAD modifier alleles at a frequency of 0.01.

Note that the invasion of FAD did not depend on its long-term effect. Specifically, FAD evolves even when it reduces mean fitness (Figure 4, corresponding to left side of Figure 2B where *c* = 0), because it reduces the mean fitness of the UNI sub-population more than that of FAD sub-populations. In this scenario the major driving force of the evolution of FAD is the *abandon-ship effect* of the modifier allele [55,56]: by increasing the dispersal rate when carrying a poor genetic background, the FAD modifier allele increases its own probability to move to a different genetic background in the next generation. That improves its chances to spread in the population by breaking away from less fit genetic backgrounds and associating with fitter ones. This advantage of the FAD modifier is “selfish” - both the individual and the population might not benefit from such a dispersal strategy that has evolved due to its selective advantage at the gene level [57].

**Figure 5:**
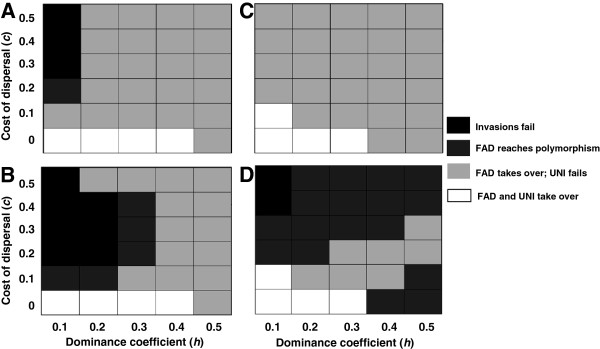
**FAD facilitates the evolution of increased dispersal rates.** Each cell shows the outcome of invasions of FAD or UNI modifier alleles. Modifier alleles are introduced at 50% to a population dispersing at a uniform rate of *α* = 0.3 (**A** and **B**) or *α* = 0.1 (**C** and **D**). If both FAD and UNI invasions were significantly more successful than expected by a neutral allele, the cell is white. If no strategy was successful, the cell is black. If only FAD was successful, the cell is light grey. If FAD was maintained at polymorphism but did not take over, the cell is dark grey. FAD facilitates the evolution of increased dispersal rates of *α* = 0.4 (**A**), *α* = 0.5 (**B**), *α* = 0.2 (**C**) and *α* = 0.3 (**D**) under a wider parameter range than UNI. The difference is particularly noticeable when there is a cost to dispersal (*c* > 0) and when the masking of deleterious alleles is not very low (*h* > 0.1). Qualitatively identical results were obtained for the introduction of FAD alleles at a frequency of 0.01 for a sample of the parameter range.

Interestingly, FAD alleles coding for increased average dispersal rate may invade a population with a uniform and lower dispersal rate, whereas similar UNI alleles cannot (Figure 5). Similarly, FAD alleles coding for decreased average dispersal rate invade populations with a uniform and higher dispersal rate under a wider parameter range than similar UNI alleles (Additional file 1). Together, these results suggest that the advantage of FAD lies in its plasticity, and not necessarily in the direction of change in dispersal rate.

Three major factors play a role in the evolution of FAD: (i) through dispersal, FAD alleles can find better genetic backgrounds, with fewer deleterious mutations overall; (ii) through dispersal, FAD alleles (as well as UNI) can find genetic backgrounds with different deleterious mutations, resulting in the masking of deleterious mutations at a heterozygote state when the wild type allele is dominant; and (iii) under FAD, the cost of dispersal is paid primarily by the less fit individuals. When invaders have a higher dispersal rate than residents and the cost of dispersal is low (including *c* ≤ 0.1, Figure 5C,D), the benefit from the masking of deleterious alleles in a heterozygote state can be higher than the costs of dispersal, and both FAD and UNI alleles can spread. However, in the absence of masking (dominance coefficient *h* = 0.5), only FAD can evolve, through efficiently associating itself with genetic backgrounds carrying fewer deleterious mutations. Similarly, intermediate masking (0.2 < *h* < 0.5), with intermediate costs (0.1 ≤ *c* ≤ 0.5), allow FAD, but not UNI, to invade. When *c* is high and *h* is low, neither FAD nor UNI can invade and increase the dispersal rate. Altogether, no single factor accounts for the evolution of FAD throughout the parameter range, with each factor being dominant in a different setting.

When FAD alleles invade a UNI population with a substantially lower dispersal rate there are cases where FAD alleles do not take over or become extinct, and polymorphism of FAD and UNI alleles - coding for lower dispersal rate - is maintained (Figure 5). This might be due to a frequency-dependent advantage of FAD alleles when they increase dispersal rates above the optimum: when the invading FAD alleles are rare, most of them occur in FAD/UNI heterozygotes, and exhibit an intermediate outcrossing rate closer to the optimum. However, FAD/UNI polymorphism is unlikely to be stable: eventually, a FAD allele coding for a more optimal dispersal frequency will appear by mutation and take over the population. For example, a FAD allele with dispersal rate *α* = 0.5 did not take over or became extinct after invading the UNI population with a dispersal rate of *α* = 0.3; but a FAD allele with dispersal rate *α* = 0.4 did take over - compare Figures 5A and 5B for *h* = 0.3 and 0.2 ≤ *c* ≤ 0.4.

Altogether, our model predicts that low quality individuals tend to disperse more often than high quality individuals. We expect the effect to be noticeable, particularly in organisms featuring both very high rates of dispersal and high costs of dispersal.

## Discussion

Our results show that Fitness-Associated Dispersal (FAD) evolves under a wide parameter range, regardless of its effect on the population mean fitness, and even in an homogeneous habitat (Figures 1, 2). Dispersal of less fit individuals can explain the evolution of high dispersal rates even under high costs of dispersal, and even when a uniform increase in dispersal rate is not favored by selection (Figure 5).

These results complement existing models showing that when the environment is heterogeneous, and mismatch between genotype and environment results in low fitness, selection favors conditional dispersal [31,37,58] such as fitness-dependent dispersal [59] or matching-habitat choice [49]. Altogether, we predict that organisms able to disperse or stay will disperse at higher rates under stress. This prediction is consistent with empirical evidence in numerous species [21,24,29].

Our model suggests that plastic dispersal can be driven by the benefits of genetic mixing, and not only of environmental change. Focusing on the genetic benefits, the model assumes conditions that are unfavorable for the evolution of dispersal in classical models: a temporally and spatially homogeneous environment, minimal kin competition, and substantial costs of dispersal. This model thus differs from classical models (reviewed in [18,32]) that assume environmental heterogeneity [60,61] and conditional dispersal [37,58] from unsuitable environments [31,62] or into suitable ones [59,63]. Our model does not assume within-deme competition. It thus differs from studies that demonstrate or require direct competition between conspecifics as a driving force for the evolution of condition-dependent dispersal (such as despotic behavior,) [64-69]. Our model can be viewed as an extension of inbreeding avoidance models for the evolution of dispersal [13-15], as we assume conditional dispersal of individuals carrying poor genomes – individuals that are more likely to experience severe inbreeding depression. The model offers unique predictions. We predict that the least fit individuals within a population will be the ones that invest more in dispersal even if there is no direct competition, and even in a homogeneous environment. However, it might be hard to exclude the possibility that individuals show this dispersal pattern because they do expect that the environment is heterogeneous, or because they do compete even when resources are currently unlimited, since they evolved in environments that on average are heterogeneous and competitive.

The concept of Fitness associated dispersal is part of the more general concept of fitness-associated genetic variation generated through mutation [70,71], recombination in a mixed population [55,72,73], and in particular costly variation, such as condition-dependent sex [56,57,74]. The marked difference between condition-dependent sex and FAD is that, in condition-dependent sex, individuals that do not meet the conditions to reproduce sexually produce offspring that are identical to themselves. However, in the case of conditional dispersal, such as FAD, individuals that do not meet the conditions to disperse essentially increase the probability to produce offspring that are inbred and may experience inbreeding depression. As a result, a gene for FAD that is linked to a good genetic background does not benefit from staying linked with that exact background (a major advantage of plasticity in previous models).

Our dispersal model may be further developed in accordance with several recent studies. First, there is no interaction among loci in this model. However, previous work has shown that FAD can facilitate adaptation even when the fitness landscape is rugged - where different mutations can be separately harmful but jointly advantageous [44]. Second, further work could test the effects of environmental heterogeneity. We would expect that environmental heterogeneity would sometimes facilitate the evolution of FAD, as dispersal is favored when either the environment is the cause of low fitness and the individual might prosper in a different environment [59], or the genotype is the cause of low fitness regardless of the environment, and changing genetic backgrounds might be advantageous, as we have shown here. In cases where the environment is heterogeneous and different genotypes match different patches in the environment, FAD can result in better genotype-to-environment matching [49,75] and accelerate local adaptation [59,76]. Third, our model assumes no temporal variation in the environment. Temporal variation often results in increased advantage for dispersal, whether uniform or plastic [12,17,58,77]. Fourth, our model assumes that the cost of dispersal is independent of condition. However, if dispersal cost decreases with condition (so that unfit individuals pay higher costs) [78] the predictions might change, possibly narrowing the parameter range allowing the invasion of FAD.

If fitness is associated with high levels of heterozygosity [79,80], our FAD model suggests that highly inbred individuals (potentially with low heterozygosity and fitness) tend to disperse at a higher rate, as demonstrated in [30]. The offspring of these dispersers are expected to show elevated levels of heterozygosity, and possibly increased fitness and decreased dispersal tendency.

Since our model does not assume that dispersal is driven by direct competition, it can naturally be extended to describe the dispersal of sessile organisms, such as plants. Plants do not typically disperse during their adult life, but rather disperse as pollen or seeds [81,82]. Thus our model has two different predictions, one for seeds and one for pollen. With respect to pollen, where gender bias is strong (i.e., mainly male gametes disperse) it was observed that maladapted hermaphroditic plants invest more in male function than in female function [83-85], usually explained through the different costs of male and female functions. Our model presents an alternative explanation for this phenomenon by suggesting that investment in pollen facilitates dispersal as well as outcrossing. With respect to seeds, all else being equal, we would also expect the further dispersing seeds to be produced more often by maladapted parent plants. Such plasticity might even help to explain the evolution of long-distance dispersal involving a high cost of dispersal through high seed mortality [86], but this topic calls for further research.

If dispersal is indeed fitness-associated in many organisms, it might have dramatic implications for evolutionary and ecological models [20]. For example, FAD in the form of matching habitat choice might change the distribution of variation across a species’ range, where clines are expected to become steeper with responsiveness of dispersal to environment, in the sense that individuals more often move out of the hybrid zone (where they may mix their gametes) in response to gradients affecting fitness [87]. FAD may further affect the stability of meta-populations, composed of core populations, where individuals are well adapted and are expected to disperse at lower rates, and peripheral populations, where individuals are expected to disperse at higher rates. FAD might thus maintain the size of core populations more stable in comparison with uniform dispersal, while allowing some gene flow of less successful individuals [88]. The pattern of dispersal may also shape biodiversity. Directed and conditional dispersal may maintain species diversity by keeping core populations more separate, while random dispersal can allow species coexistence [89].

## Conclusions

Our results show that fitness-associated dispersal (FAD), where less fit individuals are more likely to disperse, has an evolutionary advantage over non-regulated uniform dispersal, even in an homogeneous environment and in the absence of direct within-deme competition. We have demonstrated that FAD evolves regardless of its effect on the population mean fitness, because FAD modifier alleles become associated with good genetic backgrounds (*abandon-ship effect*). Thus, we suggest that plastic dispersal can be driven by the benefits of genetic mixing and not only by the benefits of environmental change. We predict that the least fit individuals in the population will disperse at the highest rates. This is expected even if environmental variability is low, individual strategy does not depend on interactions with neighbors, and dispersal carries significant costs.

## Methods

We used stochastic individual-based simulations of structured populations to study the evolutionary dynamics of a modifier gene that determines the individual dispersal strategy in a homogeneous environment. The simulated populations are constant in size and are divided into demes. Mating occurs exclusively within demes. In contrast to previous work [59], here we assume that selection against deleterious phenotypes is uniform across demes. Generations are discrete and non-overlapping, and each generation starts with dispersal, followed by reproduction and selection on the offspring.

During the dispersal stage, individuals either disperse to another deme or stay in their natal deme, depending on their dispersal strategy: uniform dispersal (UNI) or fitness-associated dispersal (FAD). UNI individuals disperse with probability *α*_*U*_, the uniform dispersal rate. In contrast, the dispersal probability of FAD individuals depends on two factors: the fitness of the individual, and the mean FAD dispersal rate *α*_*F*_. Both *α*_*U*_ and *α*_*F*_ are constant throughout a single simulation. The number of dispersing FAD individuals is randomly drawn from a binomial distribution, but their identity is determined by fitness, so that individuals with low fitness disperse first. We assume an “island model” [90], where every deme is equally close to every other deme. At the end of the dispersal stage, individuals arrive at a random deme as sexually mature adults. All dispersing individuals, independent of genotype, pay a “cost of dispersal” – they have a probability *c* (0 ≤ *c* ≤ 1) of perishing without leaving offspring due to the direct energetic costs of locomotion, predation and dispersal risks.

The genome is modeled by 10,000 diploid fitness loci and a single diploid modifier locus determining the dispersal strategy. All loci are bi-allelic: the modifier locus with UNI and FAD alleles, and fitness loci with wild-type and mutant alleles. The dispersal strategy of UNI/FAD heterozygots is set at birth, 50% of them as homozygote UNI and 50% as homozygote FAD. Segregation is Mendelian and recombination between the modifier locus and the fitness loci is free (as if they are located on separate chromosomes). The number of recombination events between fitness loci is drawn from a Poisson distribution with an expected value of 20 events per genome per generation. The number of mutations occurring at reproduction is drawn from a Poisson distribution with an expected value of 0.5 mutations per genome per generation. Mutation and recombination probabilities are uniform across the genome, and all mutations are deleterious.

Mating is strictly within-deme but is otherwise random, and all individuals are simultaneous hermaphrodites that reproduce sexually with no self-fertilization. For each new offspring, we first chose a deme with probability proportional to the deme’s relative size (see below) in order to minimize kin competition. We then chose a pair of parents from that deme to reproduce. The offspring undergo viability selection: juveniles survive to adulthood with a probability (1-*s*)^*m*^(1-*hs*)^*n*^, where *m* and *n* are the number of homozygous and heterozygous deleterious mutations in the individual’s genome, respectively, *h* is the dominance coefficient, and *s* is the selection coefficient. Surviving individuals join the next generation of their parents’ deme. This process continues until the offspring population reaches the total population size, and the parental population is then replaced by the offspring population.

We assume the environment is constant and homogeneous with respect to selection, but demes may vary in size and number between generations. Demes that are occupied by more successful genotypes tend to increase in size due to: (a) higher survival probabilities; and (b) under FAD, these demes would have more immigrants then emigrants, on average. Demes that pass a preset maximum capacity after the reproduction phase are split into two. Small demes can disappear during the reproduction phase due to random sampling, but the total population size is kept constant at 5,000. In most of the simulations the preset maximum deme capacity was 20. We performed a small number of simulations with larger deme capacities of 50 and 100, but these did not demonstrate any qualitative difference (data not shown).

We ran two types of simulations: those with populations homogeneous at the modifier loci; and those of modifier allele invasions. First, we simulated populations with an homogeneous dispersal strategy evolving to a mutation-selection balance, defined as a steady-state in population mean fitness and heterozygosity. We monitored the long-term (steady-state) effects of dispersal strategies on the population mean fitness and the fraction of deleterious alleles in a heterozygous state. We explored the following parameter range: dispersal rate *α* = 0.01, 0.1 and 0.3; cost of dispersal *c* = 0, 0.1, 0.3 and 0.5; dominance coefficient *h* = 0.2, 0.3, 0.4 and 0.5; selection coefficient *s* = 0.1. In addition, we simulated invasions of a modifier allele to a homogenous resident population at a mutation-selection balance. The invading modifier allele started at low frequency (1% or 5% of the population, without any qualitative difference). The simulation continued until one of the modifier alleles became extinct or 30,000 generations had passed. Invasion was successful if the invading modifier allele took over the population significantly more often than expected for a neutral modifier (teh expected invasion success of a neutral allele is its initial frequency, 1% or 5%). We simulated both invasions in which residents and invaders had the same dispersal rate, and invasions in which invaders had a higher dispersal rate than residents. The stimulation source code is available at https://code.google.com/p/fagr/ under a CC-BY-SA 3.0 license.

## Competing interests

The authors declare that they have no competing interests.

## Authors’ contributions

AG, AA, YR and LH designed the study. AG carried out the simulations. YR and AG wrote the simulations’ code. AG and LH performed the analyses. AG, AA, YR and LH wrote the manuscript. All authors read and approved the final manuscript.

## Supplementary Material

Additional file 1 **Figure: Invasion of FAD and UNI with decreased dispersal rates.** Each cell shows the outcome of invasions of FAD or UNI modifier alleles. **A** - Invasion with dispersal rate of 0.033 into a population with dispersal rate of 0.1; **B** - Invasion with dispersal rate of 0.066 into a population with dispersal rate of 0.1; **C** - Invasion with dispersal rate of 0.1 into a population with dispersal rate of 0.3; **D** - Invasion with dispersal rate of 0.2 into a population with dispersal rate of 0.3. Click here for file
