# Supplementary material for: Dispersing away from bad genotypes: the evolution of Fitness-Associated Dispersal (FAD) in homogeneous environments
Source: BMC Evol Biol. 2013 Jun 19;13:125. doi: 10.1186/1471-2148-13-125 (PMC3704926; doi:10.1186/1471-2148-13-125)
Supplement: Additional file 1 — Figure: Invasion of FAD and UNI with decreased dispersal rates. Each cell shows the outcome of invasions of FAD or UNI modifier alleles. A - Invasion with dispersal rate of 0.033 into a population with dispersal rate of 0.1; B - Invasion with dispersal rate of 0.066 into a population with dispersal rate of 0.1; C - Invasion with dispersal rate of 0.1 into a population with dispersal rate of 0.3; D - Invasion with dispersal rate of 0.2 into a population with dispersal rate of 0.3. [file 1471-2148-13-125-S1.ppt]

## Slide 1
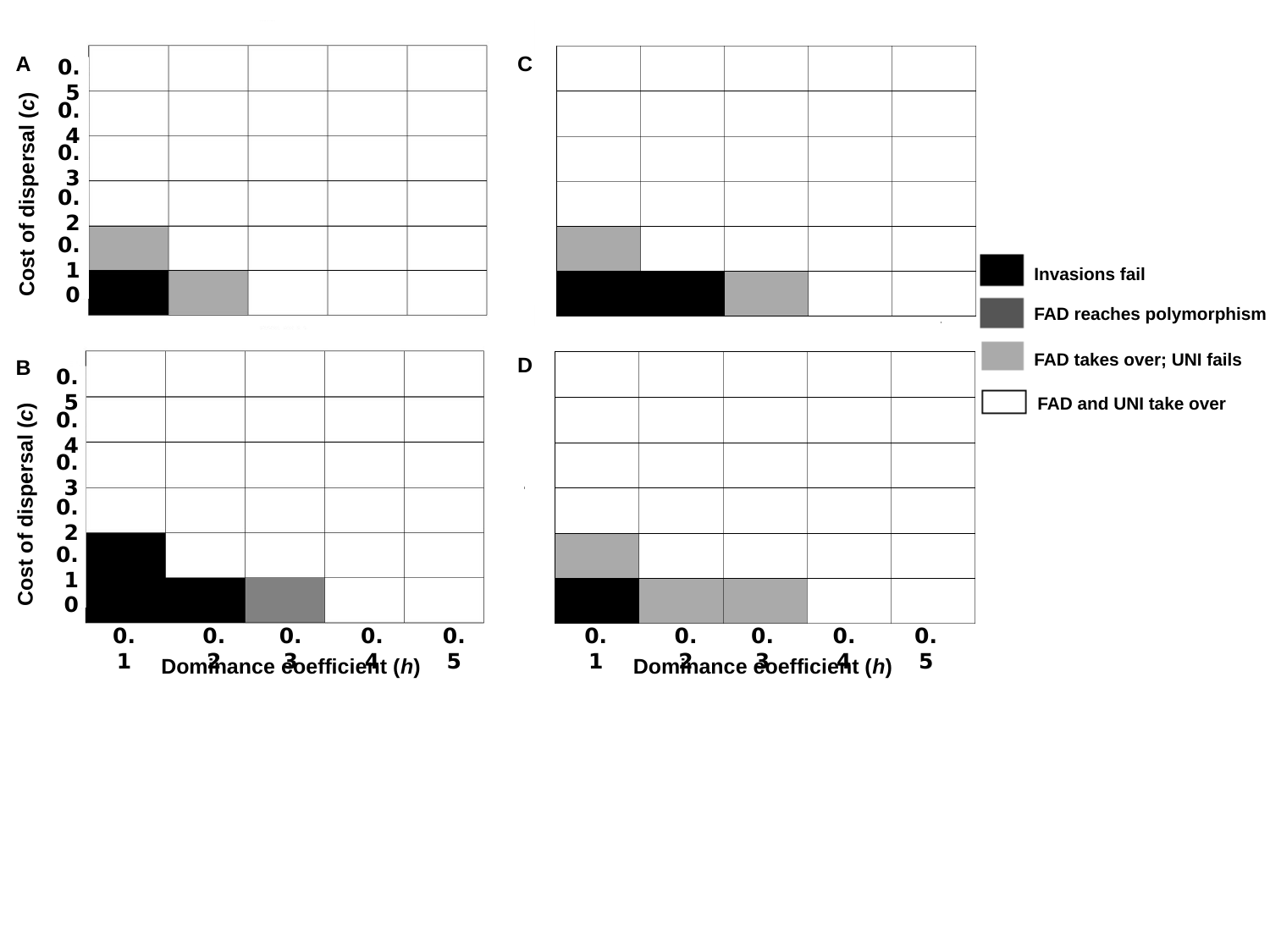

A
C
0.5
0.4
0.3
0.2
0.1
0
Cost of dispersal (c)
Invasions fail
FAD reaches polymorphism
FAD takes over; UNI fails
FAD and UNI take over
D
B
0.5
0.4
0.3
0.2
0.1
0
Cost of dispersal (c)
0.1
0.2
0.3
0.4
0.5
Dominance coefficient (h)
0.1
0.2
0.3
0.4
0.5
Dominance coefficient (h)
